# Widow Inheritance and HIV Prevalence in Bondo District, Kenya: Baseline Results from a Prospective Cohort Study

**DOI:** 10.1371/journal.pone.0014028

**Published:** 2010-11-17

**Authors:** Kawango E. Agot, Ann Vander Stoep, Melissa Tracy, Billy A. Obare, Elizabeth A. Bukusi, Jeckoniah O. Ndinya-Achola, Stephen Moses, Noel S. Weiss

**Affiliations:** 1 Department of Research, Impact Research and Development Organization, Kisumu, Kenya; 2 Department of Epidemiology, School of Public Health, University of Washington, Seattle, Washington, United States of America; 3 Department of Epidemiology, University of Michigan, Ann Arbor, Michigan, United States of America; 4 Center for Microbiology Research, Kenya Medical Research Institute, Nairobi, Kenya; 5 Department of Medical Microbiology, University of Nairobi, Nairobi, Kenya; 6 Department of Community Health Sciences and Medical Microbiology, University of Manitoba, Winnipeg, Canada; Tulane University, United States of America

## Abstract

**Background:**

Widow Inheritance is a widespread cultural practice in sub-Saharan Africa that has been postulated as contributing to risk of HIV transmission. We present baseline results from a study designed to investigate the association between widow inheritance and HIV acquisition.

**Methods and Findings:**

We performed a cross-sectional analysis of baseline data from a prospective cohort study to investigate if widow inheritance is a risk practice for HIV infection. Study participants were 1,987 widows who were interviewed regarding their inheritance status and sexual behavior profile and tested for HIV. Of these widows, 56.3% were inherited. HIV prevalence, at 63%, was similar among non-inherited and inherited widows. We stratified exposure status by the relationship of the widow to the inheritor and the reason for inheritance, and reexamined the HIV status of four subgroups of inherited women relative to the HIV status of non-inherited women. When adjusting for age and level of formal education, widows who were inherited by non-relatives for sexual ritual were significantly more likely to be infected than widows who were not inherited (OR = 2.07; 95%CI 1.49–2.86); widows who were inherited by relatives for sexual ritual also had elevated odds of HIV infection (OR = 1.34; 95%CI = 1.07–1.70). Widows who were inherited by relatives for companionship were less likely than women who were not inherited to be infected with HIV (OR = 0.85; 95%CI 0.63–1.14).

**Conclusions:**

HIV prevalence among inherited widows varied depending upon why and by whom they were inherited. The cohort study will determine the risk for HIV acquisition among the HIV seronegative widows in this sample.

## Introduction

According to the recent report of the Joint United Nations Programme on HIV/AIDS (UNAIDS) [1], the HIV/AIDS epidemic has had its most profound impact in sub-Saharan Africa, which, at the end of 2008, was home to 67.1% of people living with HIV/AIDS, 70.4% of new infections and 70% of AIDS-related deaths. Of the 31.3 million adult infections globally and 22.4 million in sub-Saharan Africa, 50.2% and 60%, respectively, occur in women. Thus, women in Africa continue to bear a much greater burden of HIV infection relative to their male counterparts, calling for targeted interventions that address women's vulnerabilities which may vary as a function of cultural and economic factors within each country and community.

In Kenya, approximately 1.4 million adults aged 15–49 years were living with HIV in 2007 [2], with a prevalence of 7.4%. The most recent Demographic and Health Survey (2008/9) [3] reports a 1.1% decline in prevalence among the same age group, to 6.3%. However, there is marked variation within the country, ranging from a high of 13.9% in Nyanza Province (16% in women and 11% in men) to a low of 0.9% in Northeastern Province [3]. Within Nyanza Province, the HIV prevalence among those identifying themselves as belonging to the Luo ethnic community was 20.2% (17.1% in men and 22.8% in women), compared to 4.7% and 7% among the neighboring Abagusii and Abaluhya communities, respectively. The prevalence of HIV among African widows has been shown to be exceptionally high. Lopman and colleagues [4], for example, reported that 61% of widows in a community sample in Zimbabwe were HIV infected. In Kenya, estimated HIV prevalence among widows was 30.2% in 2003 [5] and 43.1% in 2008/9 [3].

It has been postulated that cultural practices of widow inheritance (WI) and sexual cleansing [6]–[10], as well as non-circumcision of men [2], [3], [11], [12], are among the key factors that account for the disproportionate burden of HIV within the Kenyan Luo community. This paper focuses on WI, a traditional cultural practice where a designated male assumes responsibility for the social and economic support of a widow upon the death of her husband. The practice of WI can be categorized in terms of the type of inheritor and the purpose of inheritance. Inheritors can be brothers or cousins to the widow's late husband (brothers are traditionally preferred), or someone who is not related to the husband. Broadly, widows many enter into inheritance contract either for companionship (for sexual fulfillment as well as for social, economic and emotional support) or for sexual ritual, performed to cleanse the widow after the burial of the husband and also during rites of passage associated with birth, marriage, and death of close family members [8], [13]–[15]. Widow inheritance for purposes of companionship and support is generally long-term and either monogamous or one in which an inheritor is shared only with his wife/wives. On the other hand, widow inheritance for purposes of fulfilling a sexual ritual is generally short-term and may involve different inheritors on different occasions [6], [9]. Widows also engage in ritual sex during establishment of homes and to mark the beginning of food production seasons: cultivation, planting, and harvesting. Sexual intercourse is performed during these events to “protect” the widow and her family from experiencing adverse consequences (collectively termed *chira*) that may befall her or her family members, specifically children, grandchildren, sisters, daughters-in-law or co-wives [6], [13], [14].

In an inheritance relationship, sex plays four main roles: i) *Ritual sexual cleansing* – the Luo believe that the death of a husband confers impurity upon the widow and restricts her from participating fully in certain social events. Sexual ritual is thus performed to cleanse her and fully reintegrate her into normal community life; ii) *Bearing children* – for a young widow with children, inheritance by a brother or cousin to the husband allows for continued support by her husband's extended family. Widows without children or with few children are expected by the extended family to bear children, particularly sons, who would continue the lineage of the deceased husband; iii) *Sexual companionship* – many women are widowed young, and fulfillment of sexual desire is a major reason for being inherited; and iv), *Other sexual rituals during widowhood* – it is a societal requirement for women who are or have been married to observe a sexual ritual to mark the beginning of social events, specifically food production seasons, rites of passage, and establishment of homes [8], [9], [13]. If, through widowhood, divorce or separation a woman does not have a resident partner with whom she can carry out ritual sexual practices, she is expected to look for a man with whom to observe the sexual ritual. An inherited widow can observe this ritual with the inheritor when such occasions arise, but a widow who is not inherited has to seek another man with whom to observe the sexual ritual [6], [13].

Increasingly however, economic burdens of supporting a widow and her children, fear of HIV infection, influence from Western religions, and exposure to other cultures through education and social fora are acting in concert to cause many brothers-in-law to shun WI [6]–[8], [10], [15]. While many continue to uphold the practice, it is increasingly being equated with backwardness, and men who have attained a given social class spurn it. As a result, many widows arrange to carry out sexual rituals with non-relatives when an event occurs that requires it. The decreasing willingness of in-laws to inherit the widows has created a demand for non-relative inheritors, which has resulted in some men making a profession out of the practice by inheriting widows serially or concurrently [6]–[8]. These men who make a living from inheriting widows have come to be referred to as “professional inheritors” (*jokowiny*; sing: *jakowiny*).

We sought to estimate the prevalence of HIV infection among Luo widows in relation to the two types of the practice of WI (inheritance by a husband's relative or by a non-relative), and the two purposes of WI (inheritance for companionship or to observe a sexual ritual).

## Methods

### Ethics Statement

This study was conducted according to the principles expressed in the Declaration of Helsinki. The study was approved by the Institutional Review Board of Kenyatta National Hospital, Nairobi, Kenya, Ref # P121/10/2002. All patients provided written informed consent for the collection of samples and subsequent analysis.

The baseline HIV prevalence study of widows used a cross-sectional study design. The prevalence study was nested within a prospective cohort study that examined the association between widow inheritance and the incidence of HIV infection among the Luo ethnic community in Nyanza Province, western Kenya.

To be eligible for study participation, women must have been widowed for at least two months, not remarried at the time of enrollment, aged 18–49 years, unaware of their HIV status, and residents in Bondo District with plans to remain in the district for the two years of planned follow-up. Participants were recruited through posters, fliers, and word of mouth at meetings with community leaders, at churches, women's groups, market places, bus stops, entrances to public and private health facilities, and communal domestic water sources frequented by women. Widows who were opinion leaders were also engaged to help with recruitment, and everyone who came to the study clinic for HIV screening was requested to refer other widows to the study.

Participants agreed to be tested for HIV and other sexually transmitted infections (STIs) and to be interviewed multiple times over the course of the longitudinal study. A screening consent form was administered to eligible participants by trained female nurse-counselors fluent in the local language (Dholuo). Consented participants were administered a baseline behavioral questionnaire on socio-demographic information (e.g. age, employment, income, education, religion, marital status, duration of widowhood, and number of children), sexual behavior (e.g. number and type of sex partners, frequency of sex, use of condoms, and history of transactional and casual sex), and inheritance status. Of particular interest were the *type of inheritor* – a relative or a non-relative, and the *purpose of inheritance* – for companionship or sexual ritual.

At the end of the questionnaire administration, participants received pre-HIV test counseling, including instructions on interpretation of the test results and assessment of their preparedness to receive a positive test result. Four drops of blood were obtained from finger-prick samples, and testing was carried out using a protocol recommended by the National AIDS and STD Control Programme (NASCOP), Ministry of Health, Kenya. The test is comprised of a double, parallel, rapid test with Determine (ABBOT Japan Co., Ltd, Minato-Ku, Tokyo, Japan) and Unigold (Trinity Biotech, Bray Co. Wicklow, Ireland). Those with concordant negative or positive results were considered HIV negative or positive, respectively. After seeing and interpreting the results, participants were given post-test counseling, including discussion on a subsequent plan for risk reduction. Those with discordant results were requested to give 5 mls of venous blood for confirmation by Virostika Elisa (BioMerieux, Boxtel, The Netherlands) at the Nyanza Provincial General Hospital in Kisumu. HIV-positive widows were referred to existing post-test support groups in their neighborhoods, as well as to patient support centers at government facilities for care and treatment services. Women needing STI treatment were treated syndromically.

This paper reports on the prevalence of HIV and the practice of widow inheritance at the time of the baseline assessment for 1,987 widows who reported their inheritance status at baseline and were tested for HIV infection. Covariates considered in this analysis were those known to be related to HIV status and perceived to have direct influence on the practice of WI. For instance, women aged 15–49 years are considered to be within reproductive age hence more likely to be sexually active and inclined to inheritance [6]. Other socio-demographic characteristics examined (education, income and employment) were selected because inheritance is assumed to be more common among poor and uneducated widows who are less empowered to resist family pressure [7], [8], [10]. Religion was important because many Christian denominations condemn the practice of WI, and their members would be less likely to observe the tradition [7], [8]; residence was included because WI is more common in rural areas where family pressures are more intense [6]–[10], [14]. Finally, widows from polygamous marriages not only face family pressure to be inherited; they also face pressures from junior co-wives if they are senior in the hierarchy [6], [7].

Data analyses involved calculating the prevalence and 95% confidence interval of HIV infection and describing the practice of widow inheritance among Luo widows. Demographic, sexual, and reproductive factors were examined in relation to both widow inheritance and HIV status. The prevalence of HIV infection was calculated in non-inherited widows and in four subgroups of inherited widows: widows inherited by relatives for companionship, widows inherited by relatives for sexual ritual, widows inherited by non-relatives for companionship, and widows inherited by non-relatives for sexual ritual. Logistic regression models were fitted to determine the relative odds of HIV infection for widows in the four inheritance subgroups with uninherited widows as the reference category. We adjusted our analyses for age and educational status. Since we could not ascertain the HIV status of each widow at the time of her husband's death, it was impossible to determine the temporal sequence between infection and inheritance. However, because the average duration from HIV infection to death in Kenya during the study period was about ten years [16], [17], we conducted a sub-analysis restricted to women who had been widowed for 10 years or more. These women would be more likely to have acquired HIV infection during the course of their widowhood.

## Results

Table S1 shows descriptive features of the study sample of 1,987 Kenyan widows. The mean age of the women was 35 years (standard deviation  = 7.8 years); about two-thirds had completed primary school; 90% had monthly incomes of 1000 Kenya shillings or less; the majority were unemployed; most claimed a religious affiliation with one of a wide variety of Christian denominations; and nearly all had children.

Relevant to the specific questions addressed by this study, 56.4% reported that they were inherited, with inheritance by relative for sexual ritual being the most common type of inheritance status (27.9% of widows), followed by inheritance by non-relative for sexual ritual (12.7%), then inheritance by relative for companionship (12.6%), and lastly, inheritance by non-relative for companionship (3.2%) (Table S1). The prevalence of HIV infection was found to be 63.1% (95% CI = 60.9–65.2) in this sample, 61.7% in uninherited and 64.1% in inherited widows (OR = 1.11, 95% CI = 0.93–1.34). Stratification on the basis of type of inheritance showed the prevalence among inherited widows ranging from a low of 54.8% in widows who were inherited by a relative primarily for companionship to a high of 73.8% in widows who were inherited by a non-relative primarily for sexual ritual.

Table S1 shows that compared to HIV-negative widows, HIV- positive widows tended to: be younger in age (p<0.001), have greater educational attainment (p<0.001), be working for salary (p = 0.041), have lived fewer years in their current residence (p<0.001), not have children (p<0.001), and ranked higher in order of seniority among co-wives (p = 0.010). The average duration of widowhood among study participants was 4.3 years (SD = 4.6; IQR = 1–6). HIV status did not vary on the basis of recentness of widowhood or on sexual activity during widowhood. Nearly two thirds of both groups of women had been sexually active since becoming widows. The characteristics of uninherited widows are compared to those of inherited widows in the four inheritance categories in Table S2. Uninherited widows tended to be younger in age (p<0.001), more educated (p<0.001), more recently widowed (p<0.001), less likely to have children (p = 0.005), and less likely to have had sex with any man since their husband died (p<0.001) than inherited widows. Thus, several factors that are related to both exposure and outcome must be taken into account as potential confounders.

Results of logistic regression analyses comparing the HIV status of widows in each of the four inheritance subgroups with the HIV status of uninherited widows, unadjusted and adjusted for differences in age and educational attainment, are presented in Table 1. The relative odds of HIV infection among widows who were inherited by non-relatives for sexual ritual was more than twice that of widows who were not inherited (OR = 2.07; 95% CI = 1.49–2.86), when also adjusting for age and education. Widows inherited by relatives for sexual ritual also had an elevated odds of HIV infection (OR = 1.34; 95% CI = 1.07–1.70), whereas widows who were inherited by relatives for companionship had a lower odds of HIV infection than non-inherited widows (OR = 0.85; 95% CI = 0.63–1.14), although this was not statistically significant. Age and education were also associated with HIV status, such that odds of HIV infection were significantly reduced among older widows but were significantly elevated among widows with more formal education.

**Table 1:** Multivariable models predicting HIV-positive status among all widows (n = 1987).

	Unadjusted		Adjusted	
	OR	95% CI	OR	95% CI
**Widow inheritance status**				
Uninherited	1.00	(ref)	1.00	(ref)
Inherited by relative for companionship	0.75	0.57–1.00	0.85	0.63–1.14
Inherited by relative for sexual ritual	1.08	0.87–1.34	1.34	1.07–1.70
Inherited by non-relative for companionship	1.37	0.79–2.36	1.48	0.84–2.61
Inherited by non-relative for sexual ritual	1.75	1.28–2.39	2.07	1.49–2.86
**Age (in years)**	0.94	0.93–0.95	0.94	0.93–0.95
**Educational attainment**				
No formal education	1.00	(ref)	1.00	(ref)
Lower primary	1.68	1.16–2.45	1.41	0.96–2.08
Upper primary	2.75	1.92–3.94	1.76	1.20–2.58
Secondary	2.97	1.96–4.51	2.27	1.46–3.52

When the analysis was restricted to include only the 235 women who had been widowed for 10 years or longer (Table 2), there was a trend for widows who were inherited by a non-relative for sexual ritual purposes to have a higher likelihood of HIV infection than uninherited widows (OR = 2.36; 95% CI = 0.79–7.07).

**Table 2:** Multivariable models predicting HIV-positive status among women widowed 10 or more years ago (n = 235).

			Unadjusted		Adjusted	
	n HIV+	n HIV-	OR	95% CI	OR	95% CI
**Widow inheritance status**						
Uninherited	14	13	1.00	(ref)	1.00	(ref)
Inherited by relative for companionship	30	34	0.82	0.33–2.02	0.81	0.30–2.16
Inherited by relative for sexual ritual	53	43	1.15	0.49–2.69	1.23	0.49–3.09
Inherited by non-relative for companionship	6	3	1.86	0.38–9.00	1.96	0.39–9.87
Inherited by non-relative for sexual ritual	28	11	2.36	0.85–6.61	2.36	0.79–7.07
**Age (in years)**	**–**	**–**	0.94	0.89–0.99	0.96	0.91–1.01
**Educational attainment**						
No formal education	16	20	1.00	(ref)	1.00	(ref)
Lower primary	48	47	1.28	0.59–2.76	1.09	0.49–2.45
Upper primary	50	27	2.32	1.03–5.19	1.69	0.70–4.12
Secondary	16	10	2.00	0.72–5.59	1.58	0.53–4.75

## Discussion

In Kenya, notably among the Luo, Luhyia, Teso and Miji Kenda communities [6], [8], [15], [18], [19], as in other African countries, notably Uganda, Malawi, Zambia, Ghana, Senegal, Cote d'Ivoire, Congo and Nigeria [4], [20]–[24], the practices of widow cleansing and widow inheritance (WI) are common and viewed by many as contributing to the rapid spread of HIV in the general population. Among the Luo community, WI has been targeted for elimination by several HIV prevention campaigns [6], [8], [25]. Despite the apparent risk for HIV acquisition/transmission associated with WI, many members of the Luo community have been reluctant to relinquish this traditional practice [6]–[8], arguing that WI performed by a brother-in-law is, in fact, a strategy to check the rapid spread of HIV, because it confines a widow to one sexual partner. They maintain that it is the influx of non-relative inheritors with unknown sexual histories who practice inheritance for ritual purposes that has turned a historically safe practice into one that increases risk for HIV/STI infection.

Others argue that, regardless of the inheritor's relationship to the widow, WI is a practice that increases risk for HIV acquisition/transmission [6], [25], [26]. Several studies [4], [20]–[24], including those among the Luo of Kenya [6]–[9], [14], [15] attribute this view to a number of factors. Inheritors are almost always married and engage in concurrent sex; many widows are infected by their late husbands and are therefore likely to infect their inheritors, who would in turn infect their wives and other partners in their sexual networks. This could contribute to the rapid propagation of the infection in the general community. Condoms are rarely used in ritual sexual practices because unless seminal and vaginal fluids mix, the practice is perceived to not be properly observed. Besides Kenya [6], [9], [15], non-use of condoms by inheritors has also been reported in Uganda [20]–[22] and Zimbabwe [4], [23], [24]. A Malawian widow cleanser explained that the tradition dictates that he has sexual intercourse with the widow, then with each of his own wives, and then again with the widow, all in one night, and without a condom [23]. Furthermore, in many instances, a widow who declines to observe the practice risks being sent away from the husband's home and having her property confiscated, with little or no state protection. These widows may subsequently engage in more vulnerable risk behaviors that elevate their chances of HIV infection. Finally, the fear of HIV, coupled with exposure to education and other cultures that do not practice WI, have made brothers in-law shy away from inheriting widows of their brothers, leading to the mushrooming of professional/commercial inheritors who earn a living from inheriting multiple widows concurrently or serially. For widows not infected at the time of their husband's death, the emergence of these professional inheritors increases the possibility of HIV acquisition.

To our knowledge, these viewpoints have not been subjected to scientific investigation. This is the first epidemiological investigation of the association between HIV status and the practice of widow inheritance. Our results showed that the majority (63%) of Luo widows in our sample were HIV positive, and the majority (56.3%) participated in the practice of widow inheritance. Overall, the HIV prevalence of inherited and non-inherited widows in our study was nearly identical. However, HIV prevalence varied widely among inherited widows as a function of the reason for inheritance and relationship of the widow to the inheritor. Clues as to differences in behavioral risk between widows inherited by relatives and non-relatives are seen in Table S2, including the significantly higher proportion of women inherited by non-relatives who had had casual sex partners and sex in exchange for help since the death of their husbands. Condom use did not differentiate subgroups of widows, but instead was uniformly low (≤5%) across groups.

The claim that non-relative inheritors have introduced risk to an otherwise safe practice is supported by our observation that regardless of the duration of widowhood, inheritance by a non-relative for sexual ritual was associated with an elevated HIV prevalence. There are a number of explanations for these findings. One is that the harm reduction or increase occurs in concert with the nuances of the cultural practices. A second is that HIV infection preceded widowhood. In other words, women who would eventually become inherited by non-relatives to perform sexual rituals were those who were already HIV positive. Although study participants reported not knowing their HIV status prior to being tested by our staff, they and their extended families may have guessed based on their health status and the circumstances of the husband's death. This suspicion could, in turn, have shaped the widow and community's decision about whether and how to become inherited.

Further study in this regard is needed. We await the results of our longitudinal study of the incidence of post-widowhood infection of participants who were HIV negative at baseline. This study will allow us to establish incidence rates of HIV infection among inherited and non-inherited widows. Nonetheless, the results of this prevalence study call into question the general claim that widow inheritance places women at increased risk of HIV infection and argues for more nuanced examinations of the different ways in which this custom is practiced.

## Supporting Information

Table S1 Characteristics of sample, stratified by HIV status (n = 1987).
(0.16 MB DOC)

Table S2 Characteristics of sample, stratified by widow inheritance status (n = 1987).
(0.16 MB DOC)
