# Supplementary material for: Widow Inheritance and HIV Prevalence in Bondo District, Kenya: Baseline Results from a Prospective Cohort Study
Source: PLoS One. 2010 Nov 17;5(11):e14028. doi: 10.1371/journal.pone.0014028 (PMC2984493; doi:10.1371/journal.pone.0014028)
Supplement: Table S1 — Characteristics of sample, stratified by HIV status (n = 1987). (0.16 MB DOC) [file pone.0014028.s001.doc]

**Table S1. Characteristics of sample, stratified by HIV status (n=1987)**

|  |  |  | **HIV status** | | | | |
| --- | --- | --- | --- | --- | --- | --- | --- |
|  | Total | | HIV-positive | | HIV-negative | |  |
|  | N | % | N | % | N | % | p-value* |
| Total | 1987 |  | 1253 |  | 734 |  |  |
| **Widow inheritance status** |  |  |  |  |  |  |  |
| Uninherited | 866 | 43.6 | 534 | 42.6 | 332 | 45.2 | <0.001 |
| Inherited by relative for companionship | 250 | 12.6 | 137 | 10.9 | 113 | 15.4 |  |
| Inherited by relative for sexual ritual | 555 | 27.9 | 352 | 28.1 | 203 | 27.7 |  |
| Inherited by non-relative for companionship | 64 | 3.2 | 44 | 3.5 | 20 | 2.7 |  |
| Inherited by non-relative for sexual ritual | 252 | 12.7 | 186 | 14.8 | 66 | 9.0 |  |
| **Demographics** |  |  |  |  |  |  |  |
| Age (in years; Mean, SD) | 35.0 (7.8) | | 33.5 (7.5) | | 37.4 (7.9) | | <0.001 |
| Religious denomination |  |  |  |  |  |  |  |
| Anglican/AIC/Mennonite | 275 | 13.8 | 188 | 15.0 | 87 | 11.9 | 0.068 |
| Catholic | 423 | 21.3 | 273 | 21.8 | 150 | 20.4 |  |
| Pentecostal | 184 | 9.3 | 114 | 9.1 | 70 | 9.5 |  |
| Seventh Day Adventist | 84 | 4.2 | 56 | 4.5 | 28 | 3.8 |  |
| African Independent Churches 1 | 575 | 28.9 | 366 | 29.2 | 209 | 28.5 |  |
| African Independent Churches 2 | 253 | 12.7 | 137 | 10.9 | 116 | 15.8 |  |
| Other missionary churches | 170 | 8.6 | 105 | 8.4 | 65 | 8.9 |  |
| Other (including no religion) | 23 | 1.2 | 14 | 1.1 | 9 | 1.2 |  |
| **Socioeconomic status** |  |  |  |  |  |  |  |
| Educational attainment |  |  |  |  |  |  |  |
| No formal education | 141 | 7.1 | 61 | 4.9 | 80 | 10.9 | <0.001 |
| Lower primary | 546 | 27.5 | 307 | 24.5 | 239 | 32.6 |  |
| Upper primary | 1013 | 51.1 | 686 | 54.8 | 327 | 44.6 |  |
| Secondary | 284 | 14.3 | 197 | 15.8 | 87 | 11.9 |  |
| Employment status |  |  |  |  |  |  |  |
| Currently salaried | 32 | 1.6 | 24 | 1.9 | 8 | 1.1 | 0.041 |
| Currently self-employed | 696 | 35.0 | 459 | 36.6 | 237 | 32.3 |  |
| Currently unemployed | 1259 | 63.4 | 770 | 61.5 | 489 | 66.6 |  |
| Current occupation |  |  |  |  |  |  |  |
| None | 456 | 23.0 | 280 | 22.4 | 176 | 24.1 | 0.544 |
| Farmer | 1262 | 63.8 | 795 | 63.7 | 467 | 63.9 |  |
| Teacher | 19 | 1.0 | 15 | 1.2 | 4 | 0.6 |  |
| Medical staff | 16 | 0.8 | 10 | 0.8 | 6 | 0.8 |  |
| Business person | 193 | 9.8 | 124 | 9.9 | 69 | 9.4 |  |
| Informal | 33 | 1.7 | 24 | 1.9 | 9 | 1.2 |  |
| Monthly income (in Kenya shillings) |  |  |  |  |  |  |  |
| None | 31 | 1.6 | 17 | 1.4 | 14 | 2.0 | 0.262 |
| 1000 or less | 1743 | 89.5 | 1094 | 88.9 | 649 | 90.5 |  |
| 1001-2000 | 167 | 8.6 | 116 | 9.4 | 51 | 7.1 |  |
| 2001 or more | 7 | 0.4 | 4 | 0.3 | 3 | 0.4 |  |
| **Residence** |  |  |  |  |  |  |  |
| Number of years at present home |  |  |  |  |  |  |  |
| < 1 year | 63 | 3.4 | 42 | 3.6 | 21 | 3.0 | <0.001 |
| 1-5 years | 780 | 41.7 | 516 | 43.9 | 264 | 38.0 |  |
| 6-10 years | 441 | 23.6 | 290 | 24.7 | 151 | 21.8 |  |
| 11-15 years | 174 | 9.3 | 116 | 9.9 | 58 | 8.4 |  |
| 16+ years | 411 | 22.0 | 211 | 18.0 | 200 | 28.8 |  |
| **Marital status** |  |  |  |  |  |  |  |
| Duration of widowhood (in years; Mean, SD) | 4.3 (4.6) | | 4.1 (4.5) | | 4.5 (4.9) | | 0.066 |
| Have children |  |  |  |  |  |  |  |
| No | 101 | 5.1 | 88 | 7.0 | 13 | 1.8 | <0.001 |
| Yes | 1886 | 94.9 | 1165 | 93.0 | 721 | 98.2 |  |
| Number of co-wives shared husband with |  |  |  |  |  |  |  |
| None | 1036 | 52.2 | 672 | 53.7 | 364 | 49.6 | 0.051 |
| 1 | 633 | 31.9 | 398 | 31.8 | 235 | 32.0 |  |
| 2 | 207 | 10.4 | 124 | 9.9 | 83 | 11.3 |  |
| 3+ | 109 | 5.5 | 57 | 4.6 | 52 | 7.1 |  |
| Rank among co-wives |  |  |  |  |  |  |  |
| First | 357 | 37.6 | 234 | 40.2 | 123 | 33.4 | 0.010 |
| Second | 448 | 47.2 | 274 | 47.1 | 174 | 47.3 |  |
| Third or lower | 145 | 15.3 | 74 | 12.7 | 71 | 19.3 |  |
| **Sexual activity since husband died** |  |  |  |  |  |  |  |
| Sex with any man since husband died |  |  |  |  |  |  |  |
| No | 664 | 37.3 | 423 | 37.3 | 241 | 37.4 | 0.968 |
| Yes | 1116 | 62.7 | 712 | 62.7 | 404 | 62.6 |  |
| Any casual sex partners since husband died |  |  |  |  |  |  |  |
| No | 1703 | 95.7 | 1078 | 95.0 | 625 | 96.9 | 0.056 |
| Yes | 77 | 4.3 | 57 | 5.0 | 20 | 3.1 |  |
| Sex with any man in exchange for help since husband died |  |  |  |  |  |  |  |
| No | 1755 | 98.5 | 1117 | 98.3 | 638 | 98.8 | 0.471 |
| Yes | 27 | 1.5 | 19 | 1.7 | 8 | 1.2 |  |
| Used condoms when having sex with men since husband died |  |  |  |  |  |  |  |
| No | 1114 | 97.3 | 715 | 97.0 | 399 | 97.8 | 0.437 |
| Yes | 31 | 2.7 | 22 | 3.0 | 9 | 2.2 |  |

* p-value comparing characteristics of HIV-positive and HIV-negative widows, using chi-square tests for categorical variables and t-tests for continuous variables
