# Supplementary material for: Widow Inheritance and HIV Prevalence in Bondo District, Kenya: Baseline Results from a Prospective Cohort Study
Source: PLoS One. 2010 Nov 17;5(11):e14028. doi: 10.1371/journal.pone.0014028 (PMC2984493; doi:10.1371/journal.pone.0014028)
Supplement: Table S2 — Characteristics of sample, stratified by widow inheritance status (n = 1987). (0.16 MB DOC) [file pone.0014028.s002.doc]

**Table S2. Characteristics of sample, stratified by widow inheritance status (n=1987)**

|  | **Inheritance status** | | | | | | | | | | |
| --- | --- | --- | --- | --- | --- | --- | --- | --- | --- | --- | --- |
|  |  |  | By relative | | By relative | | By non-relative | | By non-relative | |  |
|  | Uninherited | | for companionship | | for sexual ritual | | for companionship | | for sexual ritual | |  |
|  | N | % | N | % | N | % | N | % | N | % | p-value* |
| Total | 866 | 43.6 | 250 | 12.6 | 555 | 27.9 | 64 | 3.2 | 252 | 12.7 |  |
| **HIV status** |  |  |  |  |  |  |  |  |  |  |  |
| HIV-negative | 332 | 38.3 | 113 | 45.2 | 203 | 36.6 | 20 | 31.3 | 66 | 26.2 | <0.001 |
| HIV-positive | 534 | 61.7 | 137 | 54.8 | 352 | 63.4 | 44 | 68.8 | 186 | 73.8 |  |
| **Demographics** |  |  |  |  |  |  |  |  |  |  |  |
| Age (in years; Mean, SD) | 34.0 (8.3) | | 35.1 (7.7) | | 36.5 (7.1) | | 33.8 (7.6) | | 34.9 (7.2) | | <0.001 |
| Religious denomination |  |  |  |  |  |  |  |  |  |  |  |
| Anglican/AIC/Mennonite | 146 | 16.9 | 24 | 9.6 | 62 | 11.2 | 5 | 7.8 | 38 | 15.1 | 0.053 |
| Catholic | 200 | 23.1 | 50 | 20.0 | 115 | 20.7 | 12 | 18.8 | 46 | 18.3 |  |
| Pentecostal | 80 | 9.2 | 22 | 8.8 | 50 | 9.0 | 6 | 9.4 | 26 | 10.3 |  |
| Seventh Day Adventist | 34 | 3.9 | 15 | 6.0 | 16 | 2.9 | 5 | 7.8 | 14 | 5.6 |  |
| African Independent Churches 1 | 217 | 25.1 | 78 | 31.2 | 181 | 32.6 | 18 | 28.1 | 81 | 32.1 |  |
| African Independent Churches 2 | 112 | 12.9 | 33 | 13.2 | 74 | 13.3 | 7 | 10.9 | 27 | 10.7 |  |
| Other missionary churches | 69 | 8.0 | 24 | 9.6 | 50 | 9.0 | 9 | 14.1 | 18 | 7.1 |  |
| Other (including no religion) | 8 | 0.9 | 4 | 1.6 | 7 | 1.3 | 2 | 3.1 | 2 | 0.8 |  |
| **Socioeconomic status** |  |  |  |  |  |  |  |  |  |  |  |
| Educational attainment |  |  |  |  |  |  |  |  |  |  |  |
| No formal education | 45 | 5.2 | 23 | 9.2 | 48 | 8.7 | 5 | 7.8 | 20 | 8.0 | <0.001 |
| Lower primary | 198 | 22.9 | 81 | 32.5 | 171 | 30.8 | 21 | 32.8 | 75 | 29.9 |  |
| Upper primary | 450 | 52.0 | 123 | 49.4 | 275 | 49.6 | 32 | 50.0 | 133 | 53.0 |  |
| Secondary | 172 | 19.9 | 22 | 8.8 | 61 | 11.0 | 6 | 9.4 | 23 | 9.2 |  |
| Employment status |  |  |  |  |  |  |  |  |  |  |  |
| Currently salaried | 17 | 2.0 | 2 | 0.8 | 9 | 1.6 | 0 | 0.0 | 4 | 1.6 | 0.078 |
| Currently self-employed | 273 | 31.5 | 98 | 39.2 | 219 | 39.5 | 23 | 35.9 | 83 | 32.9 |  |
| Currently unemployed | 576 | 66.5 | 150 | 60.0 | 327 | 58.9 | 41 | 64.1 | 165 | 65.5 |  |
| Current occupation |  |  |  |  |  |  |  |  |  |  |  |
| None | 210 | 24.4 | 60 | 24.3 | 109 | 19.6 | 15 | 23.8 | 62 | 24.6 | 0.108 |
| Farmer | 529 | 61.4 | 163 | 66.0 | 374 | 67.4 | 35 | 55.6 | 161 | 63.9 |  |
| Teacher | 11 | 1.3 | 0 | 0.0 | 6 | 1.1 | 0 | 0.0 | 2 | 0.8 |  |
| Medical staff | 7 | 0.8 | 1 | 0.4 | 3 | 0.5 | 0 | 0.0 | 5 | 2.0 |  |
| Business person | 91 | 10.6 | 20 | 8.1 | 51 | 9.2 | 10 | 15.9 | 21 | 8.3 |  |
| Informal | 14 | 1.6 | 3 | 1.2 | 12 | 2.2 | 3 | 4.8 | 1 | 0.4 |  |
| Monthly income (in Kenya shillings) |  |  |  |  |  |  |  |  |  |  |  |
| None | 26 | 3.1 | 0 | 0.0 | 1 | 0.2 | 0 | 0.0 | 4 | 1.6 | 0.002 |
| 1000 or less | 739 | 87.5 | 218 | 89.0 | 497 | 91.0 | 58 | 92.1 | 231 | 92.8 |  |
| 1001-2000 | 75 | 8.9 | 26 | 10.6 | 47 | 8.6 | 5 | 7.9 | 14 | 5.6 |  |
| 2001 or more | 5 | 0.6 | 1 | 0.4 | 1 | 0.2 | 0 | 0.0 | 0 | 0.0 |  |
| **Residence** |  |  |  |  |  |  |  |  |  |  |  |
| Number of years at present home |  |  |  |  |  |  |  |  |  |  |  |
| < 1 year | 28 | 3.4 | 4 | 1.7 | 20 | 3.8 | 1 | 1.6 | 10 | 4.6 | 0.598 |
| 1-5 years | 357 | 43.2 | 93 | 39.2 | 207 | 39.5 | 28 | 45.2 | 95 | 43.2 |  |
| 6-10 years | 183 | 22.2 | 66 | 27.9 | 121 | 23.1 | 13 | 21.0 | 58 | 26.4 |  |
| 11-15 years | 82 | 9.9 | 21 | 8.9 | 46 | 8.8 | 7 | 11.3 | 18 | 8.2 |  |
| 16+ years | 176 | 21.3 | 53 | 22.4 | 130 | 24.8 | 13 | 21.0 | 39 | 17.7 |  |
| **Marital status** |  |  |  |  |  |  |  |  |  |  |  |
| Duration of widowhood (in years; Mean, SD) | 1.9 (3.0) | | 7.0 (5.5) | | 5.9 (4.6) | | 5.8 (5.0) | | 5.6 (4.7) | | <0.001 |
| Have children |  |  |  |  |  |  |  |  |  |  |  |
| No | 59 | 6.8 | 10 | 4.0 | 20 | 3.6 | 6 | 9.4 | 6 | 2.4 | 0.005 |
| Yes | 807 | 93.2 | 240 | 96.0 | 535 | 96.4 | 58 | 90.6 | 246 | 97.6 |  |
| Number of co-wives shared husband with |  |  |  |  |  |  |  |  |  |  |  |
| None | 453 | 52.4 | 147 | 58.8 | 277 | 49.9 | 34 | 53.1 | 125 | 49.6 | 0.637 |
| 1 | 275 | 31.8 | 71 | 28.4 | 190 | 34.2 | 19 | 29.7 | 78 | 31.0 |  |
| 2 | 89 | 10.3 | 23 | 9.2 | 57 | 10.3 | 6 | 9.4 | 32 | 12.7 |  |
| 3+ | 47 | 5.4 | 9 | 3.6 | 31 | 5.6 | 5 | 7.8 | 17 | 6.8 |  |
| Rank among co-wives |  |  |  |  |  |  |  |  |  |  |  |
| First | 153 | 37.1 | 25 | 24.5 | 108 | 39.0 | 13 | 43.3 | 58 | 45.3 | 0.095 |
| Second | 202 | 48.9 | 59 | 57.8 | 121 | 43.7 | 13 | 43.3 | 53 | 41.4 |  |
| Third or lower | 58 | 14.0 | 18 | 17.7 | 48 | 17.3 | 4 | 13.3 | 17 | 13.3 |  |
| **Sexual activity since husband died** |  |  |  |  |  |  |  |  |  |  |  |
| Sex with any man since husband died |  |  |  |  |  |  |  |  |  |  |  |
| No | 640 | 97.1 | 2 | 0.8 | 10 | 1.8 | 3 | 4.7 | 9 | 3.6 | <0.001 |
| Yes | 19 | 2.9 | 248 | 99.2 | 545 | 98.2 | 61 | 95.3 | 243 | 96.4 |  |
| Any casual sex partners since husband died |  |  |  |  |  |  |  |  |  |  |  |
| No | 658 | 99.9 | 245 | 98.0 | 538 | 96.9 | 55 | 85.9 | 207 | 82.1 | <0.001 |
| Yes | 1 | 0.2 | 5 | 2.0 | 17 | 3.1 | 9 | 14.1 | 45 | 17.9 |  |
| Sex with any man in exchange for help since husband died |  |  |  |  |  |  |  |  |  |  |  |
| No | 662 | 99.9 | 244 | 98.4 | 543 | 97.8 | 59 | 92.2 | 247 | 98.0 | <0.001 |
| Yes | 1 | 0.2 | 4 | 1.6 | 12 | 2.2 | 5 | 7.8 | 5 | 2.0 |  |
| Used condoms when having sex with men since husband died |  |  |  |  |  |  |  |  |  |  |  |
| No | 37 | 94.9 | 233 | 95.9 | 539 | 97.8 | 63 | 98.4 | 242 | 97.6 | 0.451 |
| Yes | 2 | 5.1 | 10 | 4.1 | 12 | 2.2 | 1 | 1.6 | 6 | 2.4 |  |

* p-value comparing characteristics of widows by inheritance status, using chi-square tests for categorical variables and one-way ANOVA for continuous variables
